# Fishing trip cost modeling using generalized linear model and machine learning methods – A case study with longline fisheries in the Pacific and an application in Regulatory Impact Analysis

**DOI:** 10.1371/journal.pone.0257027

**Published:** 2021-09-07

**Authors:** Hing Ling Chan, Minling Pan

**Affiliations:** Ecosystem Sciences Division, Social-Ecological and Economic Systems Program, Pacific Islands Fisheries Science Center, National Marine Fisheries Service, Honolulu Hawaii, Hawaii, United States of America; Instituto Portugues do Mar e da Atmosfera, PORTUGAL

## Abstract

Fishing trip cost is an important element in evaluating economic performance of fisheries, assessing economic effects from fisheries management alternatives, and serving as input for ecosystem and bioeconomic modeling. However, many fisheries have limited trip-level data due to low observer coverage. This article introduces a generalized linear model (GLM) utilizing machine learning (ML) techniques to develop a modeling approach to estimate the functional forms and predict the fishing trip costs of unsampled trips. GLM with Lasso regularization and ML cross-validation of model are done simultaneously for predictor selection and evaluation of the predictive power of a model. This modeling approach is applied to estimate the trip-level fishing costs using the empirical sampled trip costs and the associated trip-level fishing operational data and vessel characteristics in the Hawaii and American Samoa longline fisheries. Using this approach to build models is particularly important when there is no strong theoretical guideline on predictor selection. Also, the modeling approach addresses the issue of skewed trip cost data and provides predictive power measurement, compared with the previous modeling efforts in trip cost estimation for the Hawaii longline fishery. As a result, fishing trip costs for all trips in the fishery can be estimated. Lastly, this study applies the estimated trip cost model to conduct an empirical analysis to evaluate the impacts on trip costs due to spatial regulations in the Hawaii longline fishery. The results show that closing the Western and Central Pacific Ocean (WCPO) could induce an average 14% increase in fishing trip costs, while the trip cost impacts of the Eastern Pacific Ocean (EPO) closures could be lower.

## Introduction

Economic data on fishing trip costs in commercial fisheries are important for effective fisheries management. They can be used to evaluate the economic performance of fisheries [1–3], estimate potential economic impacts from various conservation and management measures [4–6], and serve as input for ecosystem and bioeconomic modeling [7–10]. In the U.S. commercial fisheries, a census of trip-level information is often available for landings and fishing effort from the federal logbooks, and revenues from the commercial receipts, but not with fishing trip costs. Fishing trip costs, sometimes refer as variable costs, are costs that incurred during a fishing trip, while insurance, vessel repairs and maintenances, etc., are considered as fixed costs [11, 12]. Trip costs vary largely by trips because trip length, travel distance, gear type, etc., can be different by trips. They can also vary across vessels because vessel size and engine power affect fuel consumption [13]. Often time, trip costs were collected by ship observers, and only a sample of trip cost data were available due to limited observer coverage. The lack of individual trip cost information makes it difficult to evaluate the trip costs and profitability of individual vessels and overall fisheries. As commercial fishing behavior is largely driven by profitability, absence of cost information makes it difficult to predict fishing behavior. In addition, fisheries management alternatives may have different effects on individual or subgroups of a fishery. Without knowing the individual trip costs under different management alternatives, it is difficult to evaluate economic impacts (such as profitability) of management actions at sub-fleet level. In order to support effective fisheries management and conservation, better understanding of the trip costs for individual vessels and an entire fleet and how these costs may change in response to regulatory changes is needed. One method to address this issue is to develop modeling approach to estimate trip costs and predict trip costs of unsampled trips.

Previous research used various modeling approaches to estimate trip costs. Some studies used average costs in different units and extrapolate total trip costs, e.g. per unit of effort [7], travel distance [8], and day and travel day [14]. Some studies developed cost functions for individual cost items using ordinary least square (OLS) regressions [12] and generalized additive models (GAM) [15]. But this approach required intensive modeling by estimating cost items individually. Others studies used regressions to estimate missing trip costs using sampled trip costs. Li and Pan [16] used OLS with log transformed trip costs and trip-level and vessel-specific covariates to estimate the Hawaii longline trip costs using one year of cost-earnings survey data. Although OLS with log transformation can be used to model skewed data, studies found that retransforming the log-scale dependent variable would introduce bias [17–19], thus bias correction could become necessary. But there is no single correct method to correct bias and some methods could be labor intensive [20, 21]. To address non-normal distribution of fishing trip cost data, Das [11] and Kirkpatrick et al. [22] used GLMs with trip-level and vessel-specific covariates to estimate and predict trip costs in the U.S. northeast and Atlantic commercial fisheries, respectively. Although these previous studies used regressions to predict trip costs for unsampled trips, none of them evaluated the predictive power of the models.

To create trip cost model that can be used for cost prediction of unsampled trips and other applications, it runs into three common phenomena. First, the model needs to handle the skewed trip cost distribution as past studies found the fishing trip costs of commercial fishing fleets had skewed distributions [11, 15, 16, 22]. Second, the model needs to have good predictive power, so that unsampled and future trip costs can be predicted. Third, the modeling approach should include good predictor selection method because there is no clear guidance from literatures about what specific predictors to be included in the model that predicts trip costs. In addition, often time the predictors like trip-specific and vessel-specific variables are highly correlated. Modeling with highly correlated predicators may lead to improper selection of predictors [23] and biased estimated coefficients [24]. Therefore, it is important to use a modeling approach that can handle correlated predictors and overcome multicollinearity. These three phenomena were not fully addressed in the previous research that modeled trip costs using sampled data and predicted trip costs of unsampled trips. Therefore, our study used generalized linear model (GLM) and machine learning (ML) algorithms to estimate the functional forms of trip cost models and predict trip costs, so that the three phenomena can be fully addressed. The algorithms included 1) GLM to address the non-normal distributed trip costs and potential heteroscedasticity in the error term, 2) least absolute shrinkage and selection operator (Lasso) regularization and supervised ML to select predictors that best fit the model, avoid multicollinearity, and minimize prediction error, and 3) n-fold cross-validation techniques in ML to evaluate the predictive power of the model. We applied this modeling approach by using the sampled trip costs and the associated trip-level fishing operational data and vessel characteristics from federal logbooks in the Hawaii and American Samoa longline fisheries. Using the estimated models, we could evaluate fisheries regulatory impacts at fleet-wide, sub-fleet, and even individual vessel levels.

The two longline fisheries in this study provided a good opportunity for a case study in trip cost modeling because 1) the two fisheries used similar fishing gear (longline), so trip cost items are similar; but they targeted different tuna species and operated in different part of the Pacific Ocean, so the predictors and their effects might be different; 2) the sampled trip cost data were collected by observers for a long time series and had good coverage of the fleets, and they were representative of the population in terms of fishing area; and 3) a large selection of trip-specific and vessel-specific variables were available in the federal logbooks for all fishing trips. These provided a good basis for modeling, validating models’ predictive power, and allowing trip cost predictions for unsampled trips and an entire fleet. To evaluate the model fitting, we compared the estimated trip costs with the actual trip costs for the same sampled trips. In addition, we used the estimated model for policy analysis by predicting and comparing the trip costs for trips operated under different management actions. Knowing how trip costs changes at individual vessel level in response to management actions provides useful information regarding fishery’s resilience to current and future fisheries management alternatives. To our knowledge, no empirical study has used supervised ML and GLM with Lasso regularization simultaneously for modeling fishing trip costs. For the American Samoa longline fishery, this is the first research effort to model fishing trip costs.

## Materials and methods

GLMs are well known in fisheries research, but ML is less so. Building a model through ML approach starts with 1) using sub-set of a sample data, known as “training data”, to find an algorithm to run on the training data; 2) training the algorithm through iterative process to build the model until the algorithm reaches an acceptable level of performance (such as certain level of accuracy and precision); and 3) the resulting trained algorithm is the ML model. One of the applications of supervised ML techniques is to run regression analysis such as OLS and GLM as they target to reproduce the output value (to estimate dependent variable from a set of independent variables) from a training data and then the trained model can be used for prediction using new data.

There are several differences between ML vs. traditional econometric methods. Linear regression in traditional econometric textbooks emphasizes on obtaining unbiased estimators for a pre-defined model from economic theory but not so on model validation. ML, on the other hand, focuses on a model’s predictive power. Out-of-sample (i.e. sample not used for training) cross-validation in ML is used to assess a model’s predictive power [25, 26]. ML literature allows a bigger role of data than traditional econometrics literatures. For example, ML literature is more concerned with model over-fitting and uses regularization to prevent it, with the amount of regularization controlled by the out-of-sample predictive performance. One common form of regularization is to add a penalty term in Lasso for predictor selection [25, 27]. The advantage of using Lasso regression is the ability to perform both predictor selection and regularization that minimizes a model’s prediction error [28] and overcome multicollinearity [29, 30]. Lasso is also better than stepwise regression, the most predominant method for variable selection [31], resulting in higher explained variance [32], better variable selection and coefficient estimation [33], and out-of-sample prediction [34]. Although other machine learning algorithms such as random forests and gradient boosting can also handle large number of predictors, correlated predictors, and non-linear relationships, and GAM can handle non-linear, “wiggling” relationships between trip cost items and explanatory variables [15]; we chose to use parametric (GLM) model because it is superior in model interpretation [32] and inference [35], so that we can use the estimated models for other economic applications. The estimated coefficients in GLM are easier to interpret in both magnitude and direction of predictor effects, and they can be used to evaluate the effects from changes in covariates on trip costs. For example, when climate factors affected travel distance, the estimated coefficient for travel distance can be used to evaluate the marginal effect of a climate factor on trip costs. Unlike regression, random forests and gradient boosting are suffered from extrapolation problem because they cannot predict values outside the domain of the training dataset [35]. GAM also has limited capacity to extrapolate data [36]. This could be a problem when we want to forecast trip costs for unsampled and future trips, especially when spatial expansion has occurred in the Hawaii deep-set longline fishery [37], and could be continued in the future as tuna habitat has shifted poleward due to warmer ocean [38]. Another reason that we chose GLM over GAM was when we examined the relationships between trip costs and trip-specific variables such as trip distances and fishing days in graphical forms, they do not have wiggling relationships.

Lasso was introduced by Tibshirani [28] as a tool for subset selection. It minimizes the sum of the squared residuals in a regression model, subject to a constraint on the sum of the absolute values of coefficients, $\sum_{j=1}^{k}|\beta_{j}|\leq t$, where *t*≥0 is a tuning parameter. For GLM, it includes a random component: *Y*_*i*_ *~* N(μ, *σ*^*2*^), a systematic component: $\eta_{i}=\beta_{0}+\sum_{j=1}^{p}\beta_{j}x_{ij}$, and a link function *g* where ${{g(\mu }_{i})=\eta }_{i}=\beta_{0}+\sum_{j=1}^{p}\beta_{j}x_{ij},\;and\;E[Y_{i}]=g^{-1}(\eta_{i})$. The objective of GLM with Lasso penalty term is to solve:

$$min\;{\sum_{i=1}^{n}{(y}_{i}-\beta_{0}-\sum_{j=1}^{k}\beta_{j}x_{ij})}^{2}+\lambda \sum_{j=1}^{k}\left|\beta_{j}\right|$$ (1)

This is the same as minimizing the sum of the squared residuals plus a penalty term that penalizes on the sum of the absolute coefficients, where *λ* is the tuning/regularization parameter. When *λ* is sufficiently large, Lasso leads to solutions with some *βj* coefficients equal to zero. Iteratively Reweighted Least Squares (IRLS) is usually used to find the maximum likelihood estimates of GLM [39]. For GLM with Lasso, IRLS algorithm essentially is an iterative process that at each step new weights (*w*_*1*_, *…*, *w*_*n*_) and new working dependent variables (*z*_*1*_, *…*, *z*_*n*_) are computed, to solve the following penalized weighted least squares problem repeatedly until an optimal *λ* value is found:

$$\min\left\{{\sum_{i=1}^{n}w_{i}{(z}_{i}-\beta_{0}-\sum_{j=1}^{k}\beta_{j}x_{ij})}^{2}+\lambda \sum_{j=1}^{k}\left|\beta_{j}\right|\right\}$$ (2)

### Model estimation

Model estimation in this study involved two steps, the first step was to determine the functional forms (predictors in the models) for trip cost estimation by training ML models (using a subset of sample data as “training data”) that used Lasso regularization techniques and n-fold cross-validation approach. In this step, we included all possible covariates and ran Eq (2) using “H2O machine learning and predictive analytics” platform in R interface [40]. “H2O” platform allows running both GLM using Lasso and performing cross-validation simultaneously so that predictor selection can be performed and at the same time model performance and predictive power can be evaluated. H2O fits GLM using IRLS to find the maximum likelihood estimates of GLM and the best *λ* is selected through cross-validation performance, so that the best *λ* selects covariates that produce the lowest model prediction errors. We ran Eq (2) using different distribution and link function assumptions. In the first step, 80% of the cost data were used as the training data and 20% were used as the test data because this 80/20 division was recommended for providing a more accurate trained model [41]. Training data were used to select predictors and compare models using cross-validation to select a final model. Test data were used for unbiased assessment of the trained model’s performance [42]. For cross-validation of ML models in the first step, we can select different options like 10-fold or 20-fold cross-validation to perform. With 10-fold cross-validation on the training data, the training dataset was randomly divided into 10 equal sized subsamples. The model was fitted on 9 subsamples and the remaining one subsample was held out to compute model performance. This process was repeated 10 times and each time a different subsample was used as the validation set. Consequently, 10 cross-validation sets were produced so that every observation was used once for validation and nine times for model estimation. With 20-fold cross-validation, the training data was randomly divided into 20 equal subsamples and 20 cross-validation sets were produced and being evaluated. With higher number of folds, more training data could be used in each iteration of the cross-validation, and this would lower the bias in estimating the out-of-sample error [43]. Each of the cross-validation sets produced predictions on their subsample that was held out as the validation data (out-of-fold predictions), and model performance was being evaluated against the actual values of the validation data in terms of error metric. We applied 10-fold cross-validation to train the Hawaii model 10 times to find the best model as this is a more unbiased approach for moderate sample sizes [44, 45]. We applied 20-fold cross-validation for the American Samoa models due to the small sample size. The prediction results for training data and test data, and the cross-validation predictions will show how well the models in prediction.

In the second step of the model estimation, we used 100% of the sampled trip cost data and ran the functional form determined in the first step to estimate the model coefficients, using different distribution and link function assumptions, and OLS for comparison purposes. Models were fitted using R [46]. In this step, we also looked at the residual analysis in graphical forms for residual performance from different distribution and link function assumptions. Residual analysis was evaluated by examining plots of residuals vs. fitted values and normal probability plots of residuals (normal Q-Q plots). A model with good fit would show symmetrical distribution of residuals vs. fitted values and normal distribution of residuals. If the standardized residuals are normally distributed, the Q-Q plot will show a straight diagonal line. Finally, we determined the best model based on prediction results from the first step and residual performance in the second step.

### The fisheries

**Fig 1:**
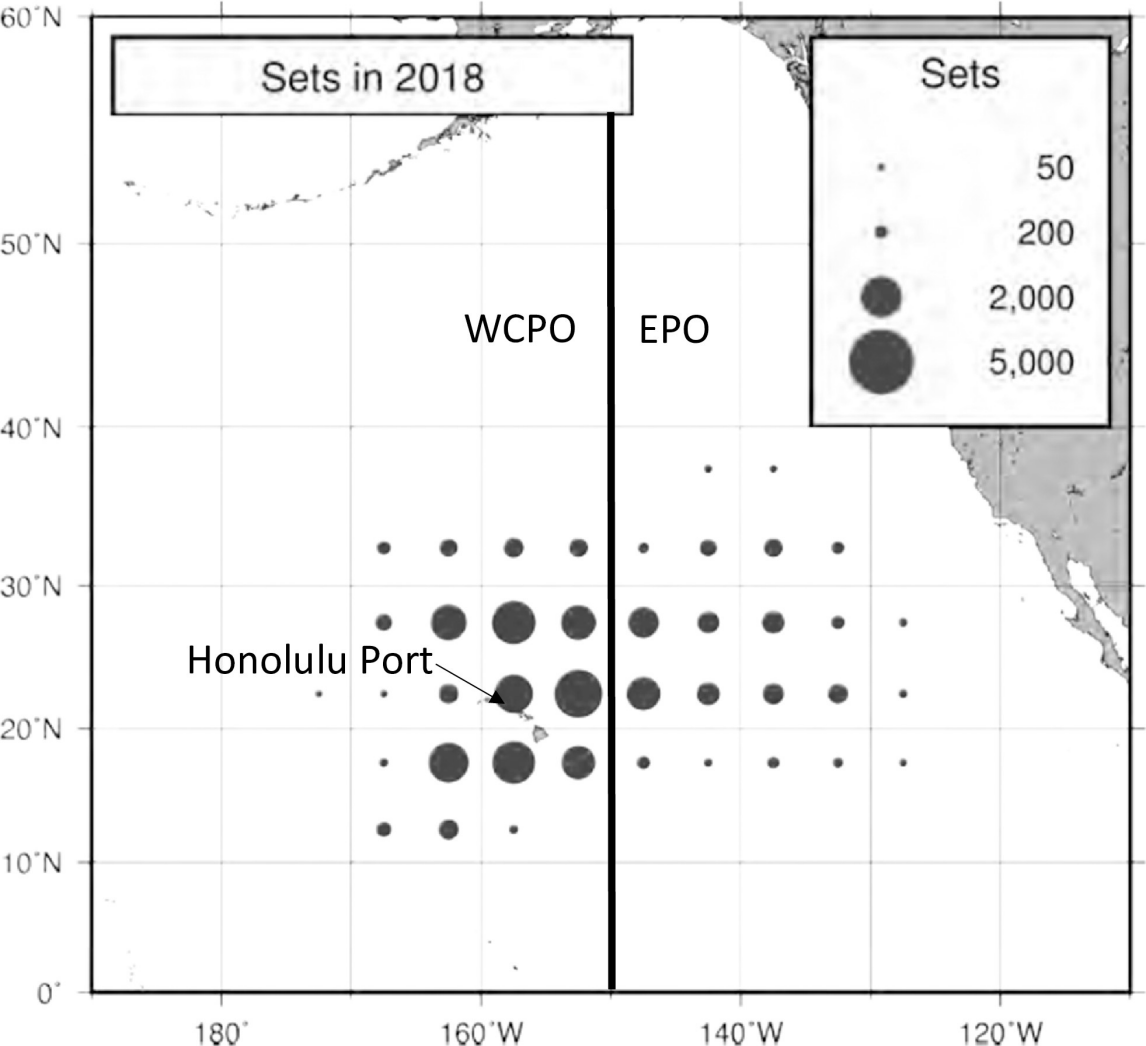
Hawaii longline fishery fishing areas: Spatial distribution of the total number of sets by longline vessels based in Hawaii and California fishing within the North Pacific Ocean, 2018 (provisional data). Source: PIFSC data report DR-12-047, https://doi.org/10.25923/dq48-ef09.

In the U.S. Pacific Island region, the Hawaii longline fishery and the American Samoa longline fishery are the two most important commercial fisheries, in both monetary support to local economies and dietary support by providing high quality of sustainable seafood. There are two segments in the Hawaii longline fishery, a deep-set longline fishery targeting bigeye tuna, and a shallow-set longline fishery targeting swordfish, with the majority of the fishing ground outside the U.S. Exclusive Economic Zone (EEZ) in the north Pacific Ocean. The fishery is managed under numerous regulations by the Western Pacific Regional Fishery Management Council (WPRFMC) including the bigeye tuna catch limits that could close certain fishing areas and consequently trip costs would be affected. The fishery is operated in two management areas: Western and Central Pacific Ocean (WCPO, west of 150°W) and Eastern Pacific Ocean (EPO, east of 150°W). Fig 1 shows the two fishing areas and the distribution of fishing effort (number of sets) in the North Pacific Ocean by the longline vessels based in Hawaii and California in 2018.

The American Samoa longline fishery is the second largest commercial fishery managed by the WPRFMC, and the fishery mainly harvests albacore tuna within EEZ in the South Pacific. Trip cost items between the two fisheries were similar because both used longline gear. These included fuel, bait, gear, provisions, ice/freezer, oil, and communication. The only extra cost item was lightsticks cost for Hawaii shallow-set trips because lightsticks were used to attract swordfish. Although the cost items were similar, the differences in trip characteristics (e.g. fishing geographic location and trip length) could have different effects on trip costs. For example, fuel cost was the main trip cost item for both fisheries that comprised more than half of the total trip costs, and bait cost was the second most important cost item that comprised around 25% and 30% of total trip costs for Hawaii and American Samoa, respectively [47]. But the differences in fishing grounds between the two fisheries (outside EEZ for Hawaii vs. inside EEZ for American Samoa) and trip length (~a month for Hawaii and 1–2 months for American Samoa) would mean that Hawaii longline trips spent more time in traveling to fishing grounds and less on fishing. Therefore, although both fisheries spent more than half of the trip costs on fuel, fuel efficiency could be different because of different travel distances to fishing grounds [48], while spending more time on fishing would result in higher percentage of bait cost in American Samoa. These differences in trip characteristics would affect the underlying predictors of trip costs.

### Covariates

Fuel cost and bait cost were the top two cost items that comprised approximately 70% of total trip costs for both Hawaii and American Samoa longline fisheries [47]; therefore, vessel-specific and trip-specific variables associated with these two cost items were incorporated into the trip cost models. Total distance traveled, average travel distance to fishing ground, trip length, fuel price, vessel length, age, gross tonnage, and net tonnage were correlated with fuel cost. Number of fishing days was associated with bait used. Fishing days might also be related to other trip cost items such as lightsticks cost, gear cost, and provision cost. The squared-term of these variables were also considered to test out the linear or nonlinear nature. Trip type (deep-set or shallow-set) was another important covariate as Kalberg and Pan [12] found substantially higher variable costs for shallow-set trips in comparison with deep-set trips in 2012. Trip type was modeled as a dummy variable in the Hawaii models. These vessel-specific and trip-specific predictors were obtained or derived from the federal logbooks [49, 50]. Details about definitions and methods to derive these predictors can be found in S1 Appendix. Tables 1 and 2 show the summary statistics of the covariates used in the final trip cost models for the Hawaii and American Samoa fisheries, respectively. For both fisheries, the trips and vessels with trip cost data had similar characteristics when compared with the overall trips and vessels. This justifies using the sampled trip cost data to model and estimate the fleet-wide trip costs.

**Table 1:** Summary statistics of the covariates in Hawaii longline fishery.

	Mean	Std. Deviation	Mean	Std. Deviation	Mean	Std. Deviation
	Trips with Fishing Costs (n = 2,746)		Trips without Fishing Costs (n = 17,161)		All Trips (n = 19,907)	
Vessel length (feet)	73.2	9.6	70.7	10.5	71.0	10.4
Vessel gross tonnage	115.1	37.7	106.9	39.9	108.1	39.7
Vessel gross tonnage per foot	1.5	0.4	1.5	0.4	1.5	0.4
Monthly fuel price ($/gallon)	4.7	0.6	4.7	0.6	4.8	0.6
Fishing days per trip	14.8	4.3	13.3	3.9	13.5	4.0
Average travel distance to fishing ground (km)	870	455	758	413	773	421
Total travel distance (km)	2,930	1,217	2,565	1,109	2,615	1,131
	Vessels with Fishing Costs (n = 167)		Vessels without Fishing Costs (n = 16)		All Vessels (n = 183)	
Vessel length (feet)	70.9	11.4	64.4	14.6	70.3	11.8
Vessel gross tonnage	107.8	43.4	85.9	51.0	105.9	44.4
Vessel gross tonnage per foot	1.5	0.4	1.2	0.6	1.4	0.5

**Table 2:** Summary statistics of the covariates in American Samoa longline fishery.

	Trips with Fishing Costs (n = 155)		Trips without Fishing Costs (n = 1,385)		All Trips (n = 1,540)	
	Mean	Std. Deviation	Mean	Std. Deviation	Mean	Std. Deviation
Vessel length (feet)	73.1	8.1	75.9	10.5	75.6	10.3
Vessel gross tonnage	113.6	33.2	121.0	39.2	120.2	38.7
Vessel gross tonnage per foot	1.5	0.3	1.6	0.4	1.6	0.4
Annual fuel price ($/gallon)	3.1	0.8	3.4	0.6	3.4	0.7
Fishing days per trip	33.4	15.8	30.6	18.1	30.8	17.9
Average travel distance to fishing ground (km)	209	116	284	219	276	212
Total travel distance (km)	2,023	1,097	2,199	1,467	2,182	1,435
	Vessels with Fishing Costs (n = 24)		Vessels without Fishing Costs (n = 5)		All Vessels (n = 29)	
Vessel length (feet)	76.8	10.5	77.9	13.8	77.0	10.9
Vessel gross tonnage	127.8	41.9	129.2	48.5	128.0	42.2
Vessel gross tonnage per foot	1.6	0.4	1.6	0.5	1.6	0.4

### Trip cost data

The trip cost data used in this study were collected through the continuous trip-level economic data collection programs at the Pacific Islands Fisheries Science Center (PIFSC) that were started in the latter part of 2004 for the Hawaii longline fishery and in 2006 for the American Samoa longline fishery. Trip cost data were collected by observers at sea during the observed fishing trips. The average observer coverage rates were 25% and 18% in Hawaii and American Samoa, respectively [47].

A total of 2,948 trips were observed and gathered trip cost data in the Hawaii longline fishery between 2005 (the first year with cost data collected for the whole year) and 2018. Some observations were excluded from model estimation including trips with some missing cost items (n = 119), outliers (n = 64), and missing logbook data (n = 19). There were 164 trips with trip cost data in the American Samoa longline fishery between 2006 and 2018, and 19 outliers were excluded. The final dataset for analysis included 2,746 trips for Hawaii for 2005–2018 (13.8% of the 19,907 total fishing trips) and 155 trips for American Samoa for 2006–2018 (10% of the 1,540 total fishing trips) (S1 Table).

Although the portions of fishing trips with trip cost data were low, the trip cost data used for modeling had extensive coverage of vessels in the fleets; therefore, there was no bias in certain types of vessels missing trip cost data. The Hawaii trip cost data used for modeling covered 91% of active vessels (167 vessels) between 2005 and 2018, and these 167 vessels represented 99% of all fishing trips in the study period. For American Samoa, the trip cost data covered a large majority of active vessels (83%, n = 24) between 2006 and 2018, and these 24 sampled vessels represented 93% of all fishing trips in the study period.

Hawaii fishing trip costs were found to be different by fishing area due to differences in travel distance, and more apparent for deep-set trips than shallow-set trips [47]. In order to determine how well the trip cost data generalized to the population in terms of the distribution of trips across different fishing areas, one sample chi-square tests were conducted for both trip types (Table 3). The test results showed that the distribution of trips by fishing area in the sample was representative of all trips (*χ*^*2*^ = 0.297, *p* = 0.862 for deep-set trips and *χ*^*2*^ = 0.854, *p* = 0.653 for shallow-set trips), as *p* value > 0.05 indicated that the null hypothesis of the equality of proportions in sample and population was not rejected. The chi-square test results and the extensive coverage of vessels in the Hawaii longline fleet supported the use of the estimated trip cost model to extrapolate unsampled trips.

**Table 3:** Distribution of trips by fishing area, and chi-square results for trips by fishing area with sample and population proportion, for deep-set and shallow-set trips.

	Sample deep-set	Population deep-set	Sample shallow-set	Population shallow-set
Trips fished in WCPO only	77.7%	77.7%	57.5%	57.1%
Trips fished in EPO only	9.1%	9.4%	23.3%	24.7%
Trips fished in both WCPO & EPO	13.2%	12.9%	19.2%	18.3%
Total number of trips	2,068	18,894	678	1,013
*χ*^*2*^ (df = 2)	0.297 (*p* = 0.862)		0.854 (*p* = 0.653)	

**Fig 2:**
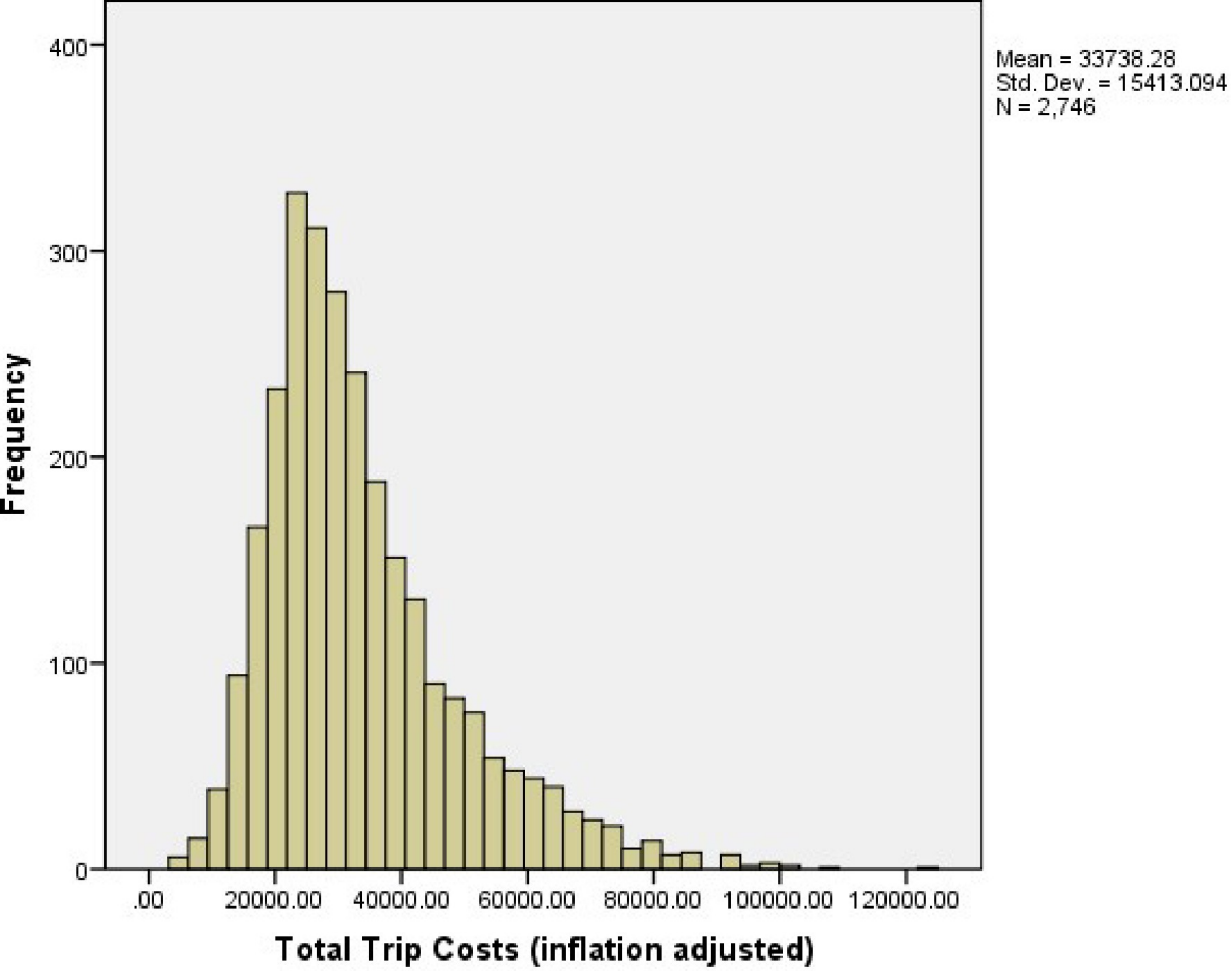
Histogram of total trip costs for sampled trips in Hawaii longline fishery: 2005–2018.

**Fig 3:**
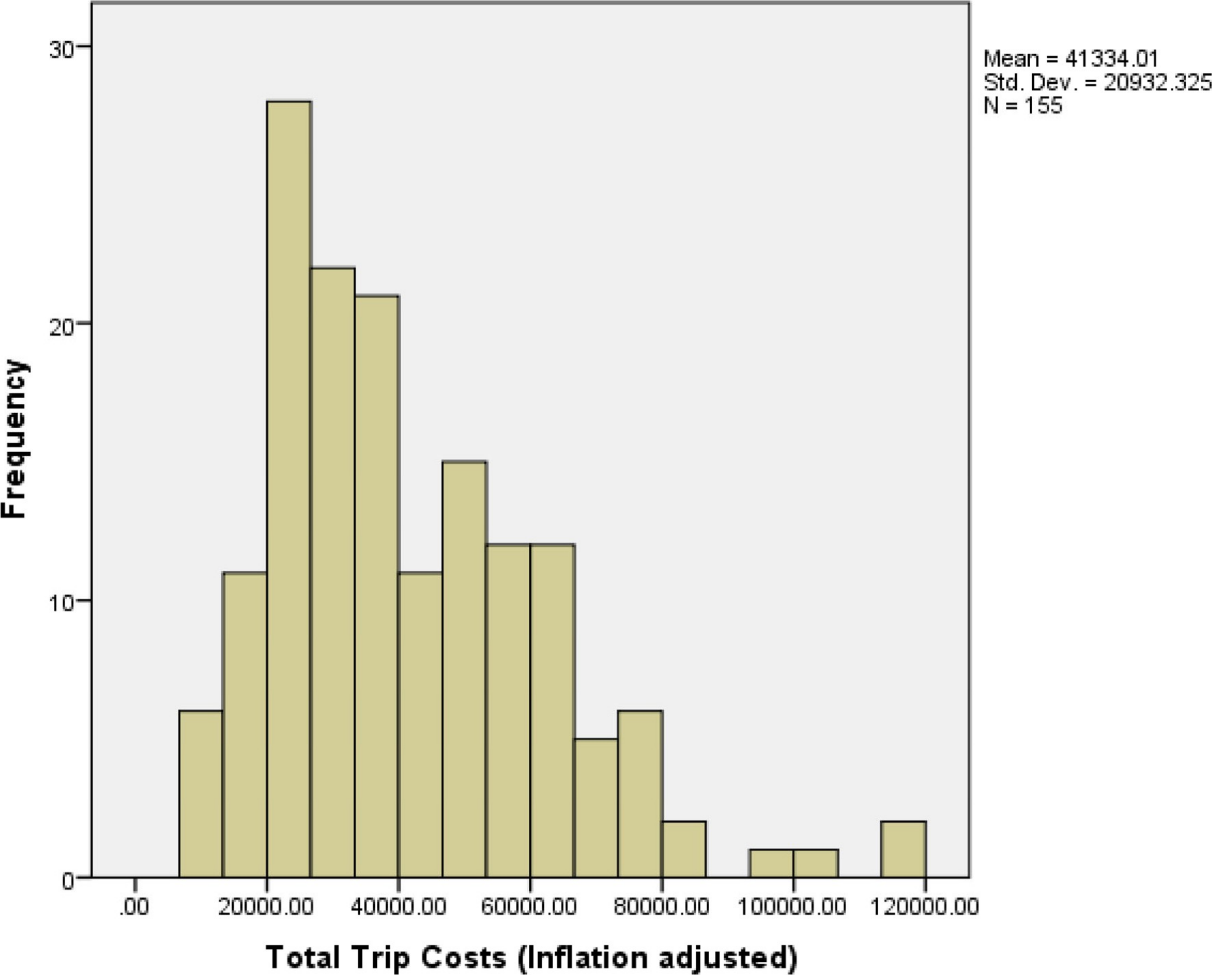
Histogram of total trip costs for sampled trips in American Samoa longline fishery: 2006–2018.

Figs 2 and 3 display the inflation adjusted (to 2018 dollar values) trip cost distributions for the sampled trips that were used for analysis in the Hawaii and American Samoa longline fisheries, respectively. The average trip cost was $33,738 in Hawaii and $41,334 in American Samoa.

Both Figs 2 and 3 show that the cost distributions for the sampled trips are not normal but rather have a fat tail on the right. This may be due to the large variances of travel distances for both fisheries and the two-gear type usage in the Hawaii longline fishery. S2 Appendix shows the statistical tests for trip cost distributions in Hawaii and American Samoa, and the results show that both distributions are skewed right.

### Policy variables

The Hawaii longline fishery had experienced short-term area closures five times in the WCPO and five times in the EPO between 2005 and 2018 [51]. However, the area closures were applicable to certain types of vessels. When the bigeye catch limit in EPO was reached, the EPO was closed only to the vessels greater than 24 feet in vessel length, while smaller vessels were still allowed to fish in the EPO. When the bigeye catch limit in WCPO was reached, the WCPO was closed only to the vessels with Hawaii longline permit and they had to travel to further area to fish, while the vessels with dual permits (with both Hawaii and American longline permits) were still allowed to fish in the WCPO. Thus, different groups of vessels may react differently to the area closures [51, 52]. During the area closures, fishermen could choose to fish in a different area or not to fish. In the Hawaii longline fishery, fishermen often chose to continue tuna fishing in a different area during area closures instead of changing the gear type to target swordfish because of the additional costs to switch to shallow-set fishing. Also, catch limits were always reached in the latter part of the year when it was not the season for shallow-set fishing. When fishermen traveled to a different fishing ground to fish, the travel distance and fishing days might change. These behavior changes were captured in the trip cost model because travel distance and fishing days were included as predictors in the trip cost model. Using the trip cost model to estimate trip costs of different subgroups, we can evaluate the trip cost effects due to area closures.

## Results

When using different distribution and link function assumptions to run Eq (2), the same functional form was found. The functional form of the ML model for Hawaii longline fishery trip costs is:

$$\begin{array}{l} g(Adjusted\;trip\;cost_{ij})=\beta_{0}+\beta_{1}\;Trip\;Type_{ij}+\beta_{2}\;Total\;Travel\;Distance_{ij}+\\ \;\beta_{3}\;Total\;Travel\;Distance_{ij}{}^{2}+\beta_{4}\;Monthly\;Fuel\;Price+\beta_{5}\;Fishing\;Days_{ij}+\\ \;\beta_{6}\;Fishing\;Days_{ij}{}^{2}+\beta_{7}\;Vessel\;Length_{j}+\beta_{8}\;Vessel\;Gross\;Tonnage_{j}+\\ \;\beta_{9}\;Vessel\;Gross\;Tonnage_{j}{}^{2}+\varepsilon_{ij}. \end{array}$$ (3)

The functional form of the ML model for American Samoa longline fishery trip costs is:

$$\begin{array}{l} g(Adjusted\;trip\;cost_{ij})=\beta_{0}+\beta_{1}\;Average\;Travel\;Distance\;to\;Fishing\;Ground_{ij}+\\ \;\beta_{2}{\left(Annual\;Fuel\;Price\right)}^{1/2}+\beta_{3}\;Fishing\;Days_{ij}+\beta_{4}\;Fishing\;Days_{ij}{}^{2}+\\ \;\beta_{5}\;Vessel\;Gross\;Tonnage\;Per\;Foot_{j}+\varepsilon_{ij}, \end{array}$$ (4)

where *i* stands for individual fishing trip and *j* stands for individual vessel, and *Trip Type*_*ij*_ = 1 for shallow-set trips and *Trip Type*_*ij*_ = 0 for deep-set trips in the Hawaii models.

Tables 4 and 5 show the prediction results from the ML models for the training data and test data and the cross-validation model results for Hawaii and American Samoa, respectively. Both tables show that for all models, the root mean squared error (RMSE) and mean absolute error (MAE) decreased when the models were scored on the test data, indicating overfitting did not occur and the models had good predictive power. For Hawaii models, Gaussian with log link had the lowest errors in the training data whereas gamma with log link had better prediction results in the test data. For American Samoa models, Gaussian with log link had the best prediction results. Multicollinearity of covariates was checked using Variance Inflation Factor (VIF). The VIF values for all covariates (excluding the squared terms) in the estimation models were less than 4 for the Hawaii models and around 1 for the American Samoa models, indicating no multicollinearity.

**Table 4:** Machine learning model prediction results for Hawaii longline fishery tip cost models.

	Gaussian with identity link	Gaussian with log link	Gamma with log link
Prediction results for the training data, *n* = 2,196			
RMSE	7,527	7,174	7,186
MAE	5,486	5,222	5,229
*R* ^*2*^	0.765	0.787	0.786
Prediction results for the test data, *n* = 550			
RMSE	7,141	7,046	7,036
MAE	5,254	5,137	5,126
*R* ^*2*^	0.770	0.776	0.777
10-fold cross-validation prediction results for the training data, *n* = 2,196			
RMSE	7,588	7,239	7,237
MAE	5,524	5,260	5,260
*R* ^*2*^	0.761	0.783	0.783

**Table 5:** Machine learning model prediction results for American Samoa longline fishery tip cost models.

	Gaussian with identity link	Gaussian with log link	Gamma with log link
Prediction results for the training data, *n* = 124			
RMSE	11,205	10,950	11,250
MAE	8,477	8,167	8,473
*R* ^*2*^	0.734	0.746	0.732
Prediction results for the test data, *n* = 31			
RMSE	9,421	8,881	10,225
MAE	7,553	7,111	8,036
*R* ^*2*^	0.691	0.725	0.636
20-fold cross-validation prediction results for the training data, *n* = 124			
RMSE	11,799	11,570	12,335
MAE	8,925	8,665	9,257
*R* ^*2*^	0.705	0.717	0.678

The results show that the functional forms for trip cost estimation for the Hawaii longline (3) and American Samoa longline (4) are similar, but with two differences. One difference is total travel distance and its squared term are important covariates in the Hawaii models whereas average travel distance to fishing ground is an important covariate in the American Samoa models. This could be due to the different trip characteristics as shown in Tables 1 and 2. The other difference is the American Samoa models perform better with vessel gross tonnage per foot, whereas the Hawaii trip cost models perform better with gross tonnage and its squared term. The negative coefficient of the squared gross tonnage indicates that the Hawaii longline trip costs are increasing at a decreasing rate in relation with gross tonnage. The difference in fishing operational areas that affected the travel distance to fishing ground could cause the different economies of scale from vessel size and capacity.

**Fig 4:**
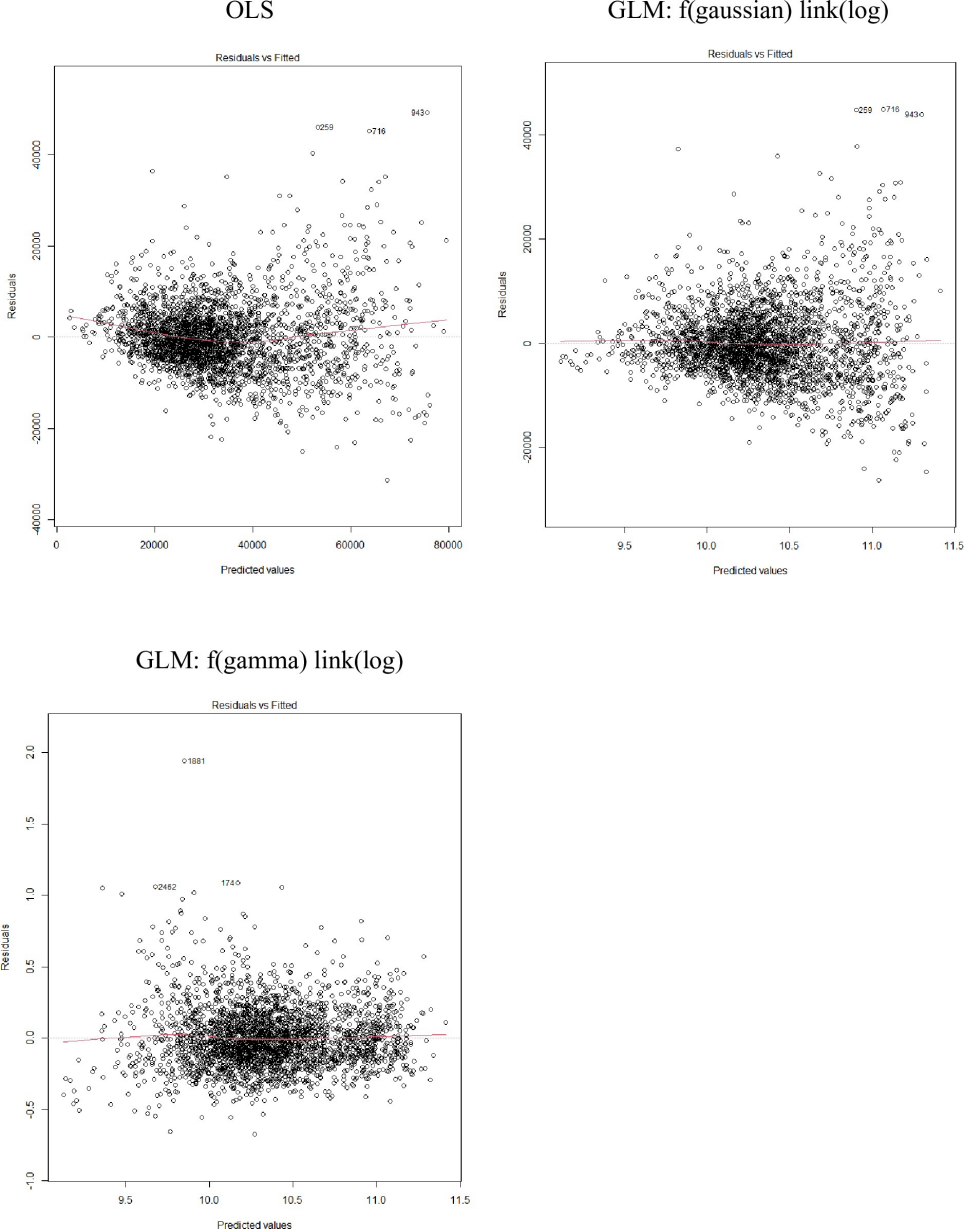
Residuals vs. fitted values plots for Hawaii longline fishery trip cost models*. * Residuals represent deviance residuals for GLM. The red line represents the average value of the residuals at each value of fitted value.

**Fig 5:**
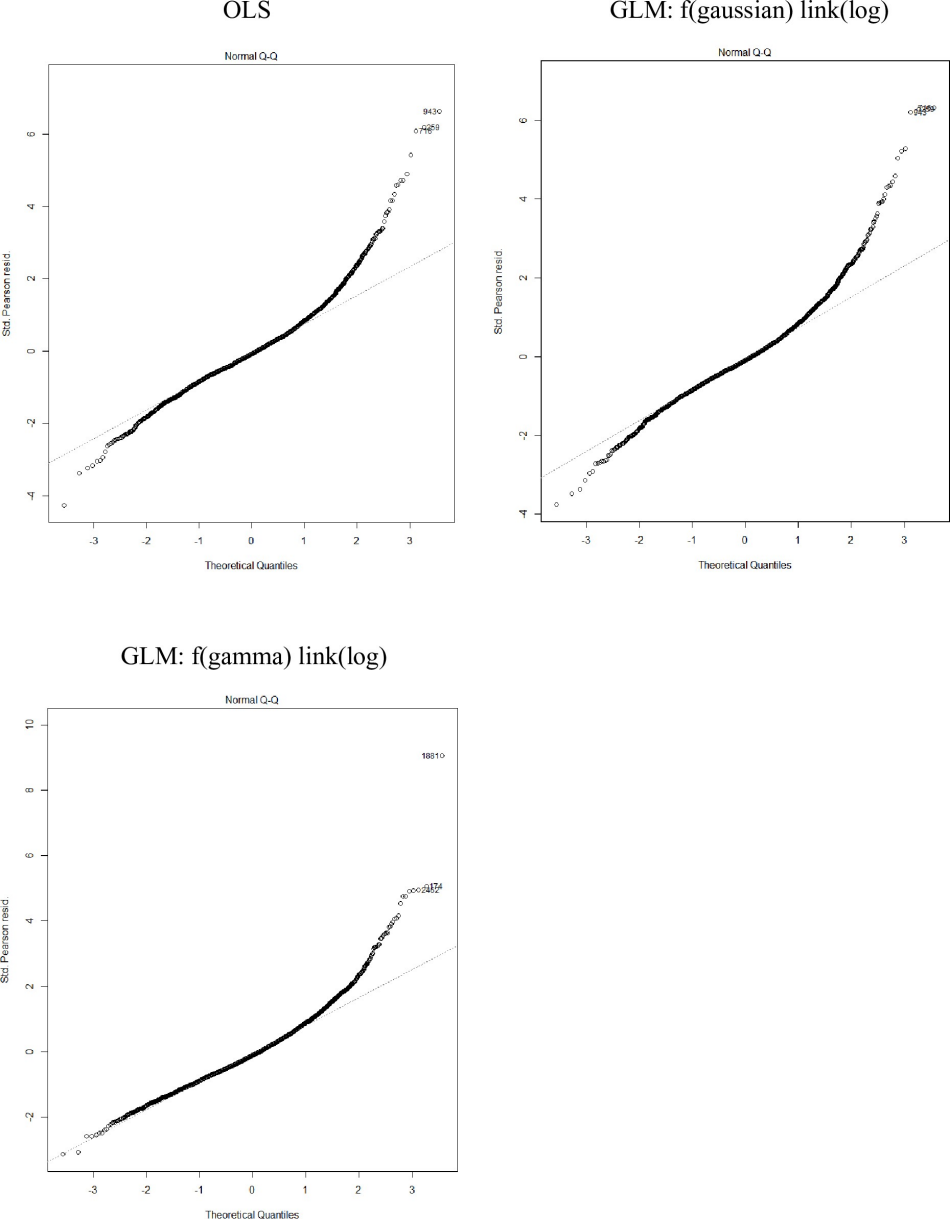
Q-Q plots for standardized deviance residuals for Hawaii longline fishery trip cost models.

Using the entire available trip cost data and the machine training models, we estimated the coefficients of the models. Table 6 shows the model results for Hawaii longline trip costs. Residuals vs. fitted values plots and Q-Q plots show that gamma with log link model performs better. Residuals from OLS model and Gaussian with log link model exhibit heteroscedasticity (Figs 4 and 5). We consider gamma with log link the best model because of the symmetric distribution of the residuals and the more normally distributed standardized residuals, and also the better prediction performance in the test data in step 1.

**Table 6:** Model results for Hawaii longline fishery tip costs.

	OLS	Gaussian with log link	Gamma with log link
Trip type	13,284.08**	0.3257**	0.3298**
	(31.91)	(32.95)	(27.50)
Total travel distance (km)	0.46	0.0001**	0.0001**
	(0.94)	(9.25)	(7.77)
Total travel distance^2^ (km)	0.0004**	-0.00000001**	-0.000000005*
	(5.00)	(-4.27)	(-2.24)
Monthly fuel price	5,632.59**	0.1622**	0.1693**
	(21.92)	(23.94)	(22.88)
Fishing days	459.25**	0.0482**	0.0570**
	(2.94)	(10.48)	(12.68)
Fishing days^2^	20.87**	-0.0006**	-0.0009**
	(4.13)	(-4.74)	(-6.13)
Vessel length (feet)	219.29**	0.0075**	0.0072**
	(7.10)	(8.71)	(8.08)
Vessel gross tonnage	80.66**	0.0064**	0.0061**
	(3.42)	(8.24)	(9.05)
Vessel gross tonnage^2^	-0.23**	-0.000021**	-0.000021**
	(-2.64)	(-7.90)	(-8.27)
Constant	-35,260.74**	7.6447**	7.6210**
	(-16.48)	(108.85)	(123.64)
Observations	2,746	2,746	2,746
AIC	56,773	56,548	55,955
RMSE	7,438	7,140	7,158
MAE	5,428	5,194	5,200
*R* ^*2*^	0.767	0.785	0.775

Note.—Numbers in parenthesis are t-ratios

* significant at the 5% level

** significant at the 1% level.

**Fig 6:**
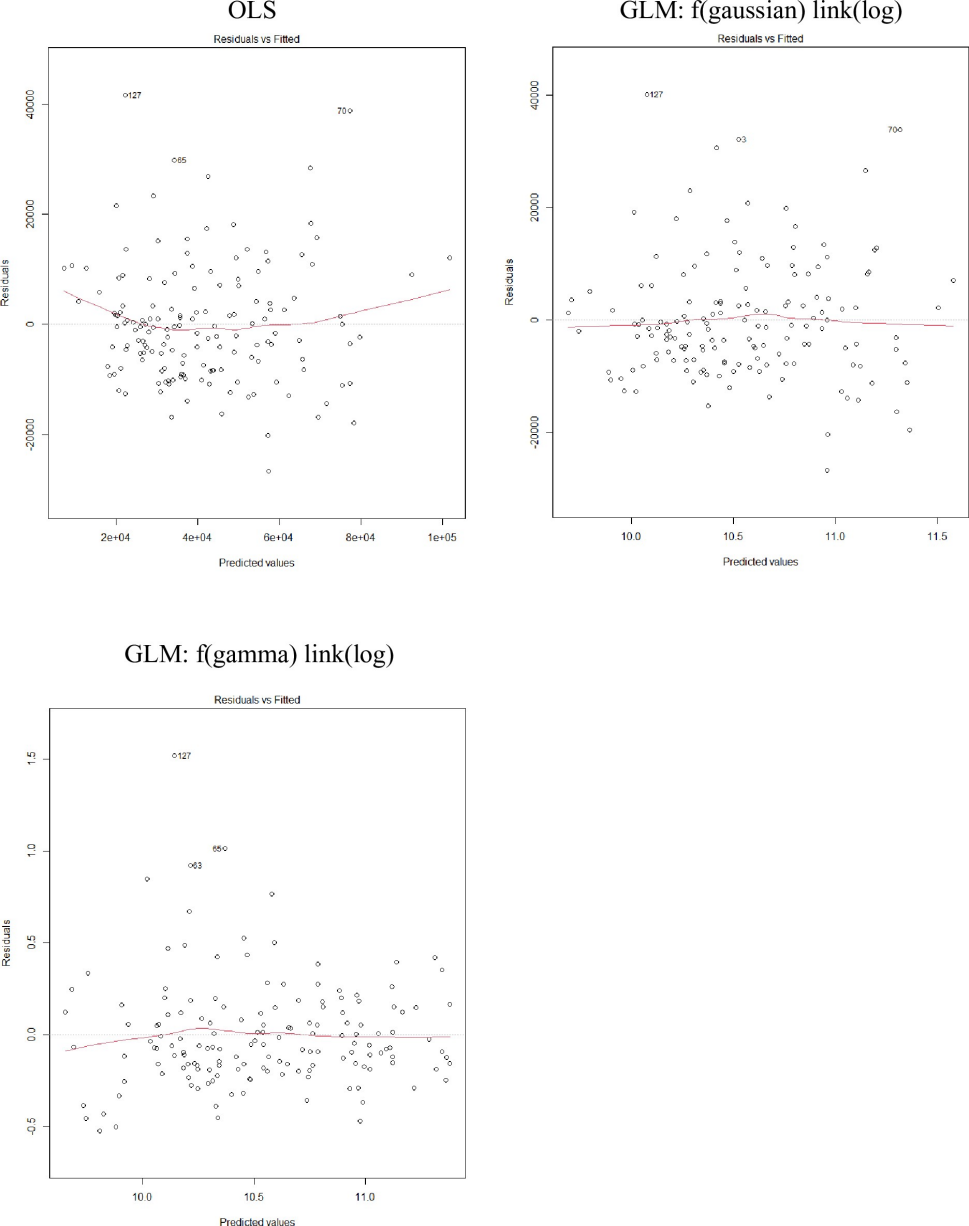
Residuals vs. fitted values plots for various American Samoa longline fishery trip cost models*. * Residuals represent deviance residuals for GLM. The red line represents the average value of the residuals at each value of fitted value.

**Fig 7:**
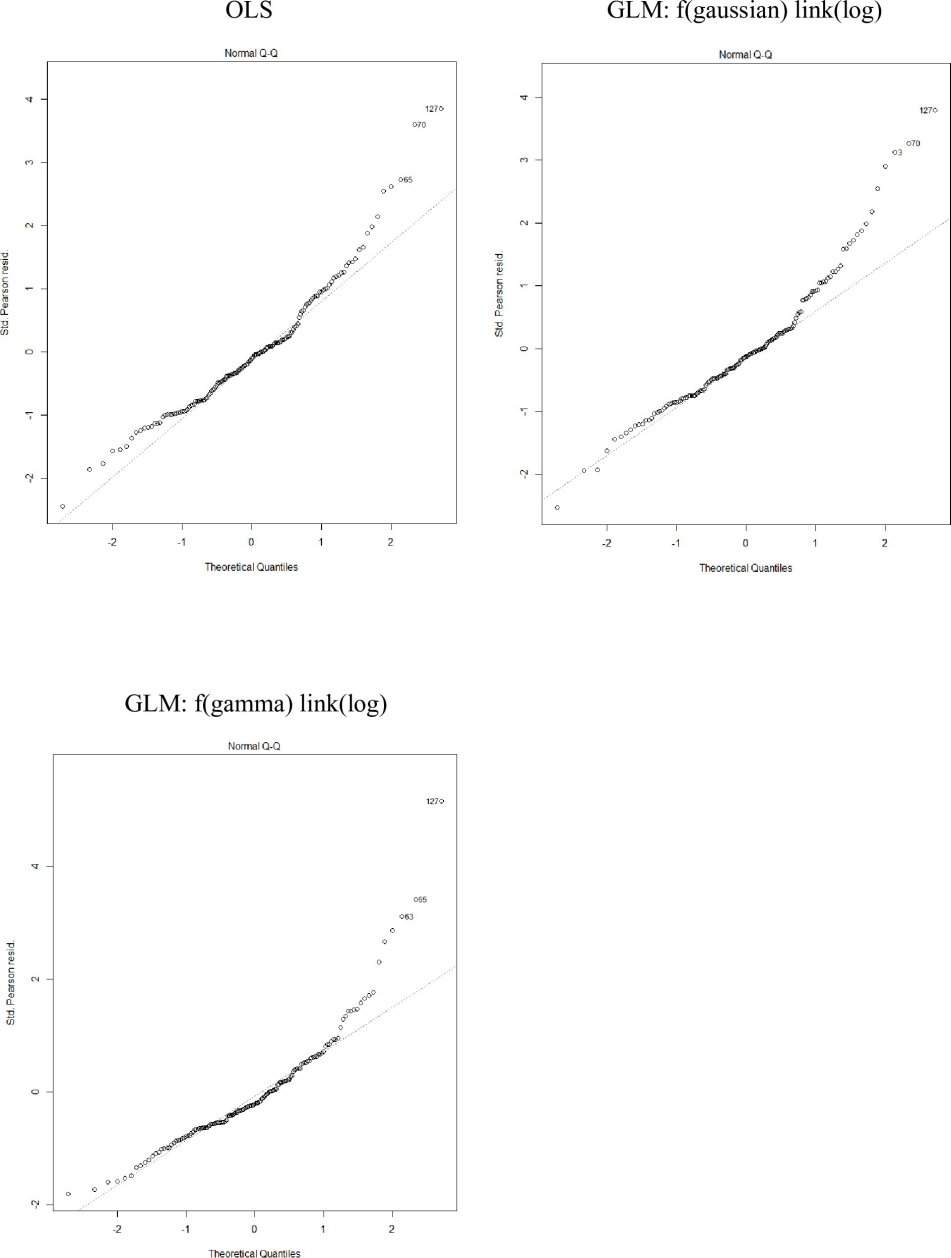
Q-Q plots for standardized deviance residuals for various American Samoa longline fishery trip cost models.

Table 7 shows the model results for American Samoa longline trip costs. Residuals vs. fitted values plots show a more symmetric distributed residuals in gamma with log link model (Fig 6), and Q-Q plots show gamma with log link model performs slightly better (Fig 7). It is hard to determine the best model because Gaussian with log link performed the best in prediction but gamma with log link showed better distribution of residuals.

**Table 7:** Model results for American Samoa longline fishery tip costs.

	OLS	Gaussian with log link	Gamma with log link
Travel distance to fishing ground (km)	12.10	0.0003*	0.0003
	(1.55)	(1.98)	(1.44)
Annual fuel price^1/2^	25,357.48**	0.6581**	0.4998**
	(6.47)	(7.47)	(4.69)
Fishing days	961.71**	0.0343**	0.0445**
	(4.61)	(7.22)	(7.84)
Fishing days^2^	-0.13	-0.0002**	-0.0003**
	(-0.05)	(-3.45)	(-4.05)
Vessel gross tonnage per foot	11,145.03**	0.2046**	0.3063**
	(4.14)	(3.28)	(4.19)
Constant	-54,643.33**	8.0915**	8.0358**
	(-6.16)	(39.36)	(33.30)
Observations	155	155	155
AIC	3,333	3,323	3,310
RMSE	10,788	10,455	10,947
MAE	8,100	7,722	8,126
*R* ^*2*^	0.733	0.749	0.706

Note.—Numbers in parenthesis are t-ratios

* significant at the 5% level

** significant at the 1% level.

### Model fitting

To examine the model fitting, we compared the estimated trip costs from the models with the actual trip costs for the same sampled trips (Table 8). For Hawaii, the GLM that produced the best model results, gamma with log link, was used for cost estimation. The average estimated trip cost for the sampled Hawaii longline trips was $33,725, which was almost the same as the actual trip cost ($33,738). The model also estimated well for both deep-set and shallow-set trips.

**Table 8:** Estimated and actual trip costs for sampled trips in Hawaii longline fishery.

	Estimated trip costs from Gamma with log link for sampled trips	Actual trip costs for sampled trips
All Trip Type		
Mean	33,725	33,738
Std. Deviation	13,508	15,413
N	2,746	2,746
Deep-set Trips		
Mean	27,710	27,668
Std. Deviation	7,188	8,980
N	2,068	2,068
Shallow-set Trips		
Mean	52,072	52,255
Std. Deviation	11,597	16,159
N	678	678

For American Samoa, the model fitting results for both Gaussian with log link and gamma with log link models are presented in Table 9. The estimated trip costs for sampled trips from the two models were very close to the actual trip costs (-0.3% difference).

**Table 9:** Estimated and actual trip costs for sampled trips in American Samoa longline fishery.

	Estimated trip costs from Gaussian with log link for sampled trips	Estimated trip costs from Gamma with log link for sampled trips	Actual trip costs for sampled trips
Mean	41,235	41,195	41,334
Std. Deviation	17,833	17,774	20,932
N	155	155	155

### Policy analysis (Regulatory Impact Analysis)

In this section, we examined the cost impacts of area closures due to reaching the annual bigeye tuna catch limits to the Hawaii longline fleet. We applied the Hawaii trip cost model (gamma with log link) and used the trip operational data and vessel characteristics for all deep-set trips (target bigeye tuna) to estimate the trip costs for all deep-set trips in the entire study period between 2005 and 2018. We tested if the fishery closures affected the cost function structure by adding two dummy variables that represented trips affected by the WCPO and EPO closures in the estimated trip cost model and the results showed that the dummy variables were not significant. This validated the use of the estimated Hawaii trip cost model for this policy analysis.

Among all the Hawaii deep-set longline trips between 2005 and 2018 (18,894 trips), five trip types were identified (Table 10). The first three types were under normal operations without any closures, these included trips that: 1) only fished within WCPO (76.5%), 2) fished in both WCPO and EPO within one trip (12.9%), 3) only fished within EPO (7.7%). The other two types included 4) trips within EPO by the affected vessels during the WCPO closures (1.7%), and 5) trips within WCPO by the affected vessels during the EPO closures (1.2%). Although only a small percentage of trips (2.9%) were affected by the closures, but the percent of affected vessels was high (73%). For trips that were taken by the affected vessels during the WCPO closures (type 4), their travel distances and fishing days were significantly longer than the regular trips that fished exclusively in the WCPO (type 1), the area where vessels were most likely to fish without the WCPO closures. The behavior of increasing fishing effort during the WCPO closures was consistent with Chan [53] and Mangi et al. [54] in the way that vessels used to fish inside the closed areas had increased their fishing effort after the marine protected area closures. On the other hand, for vessels that were affected during the EPO closures (type 5), their trips had the shortest travel distances and longest fishing days. The average trip costs for each of the trip types are shown in Table 10. For the trips taken by the affected vessels during the WCPO closures (type 4), the average trip cost ($29,092) was higher than the regular WCPO trips (type 1) because of the longer travel distances (+1,667 km) and fishing days (+1 day). Therefore, if the bigeye catch limit in WCPO reduced further, we could expect the trip cost to increase by 14% on average, as affected vessels have to move to the EPO, otherwise stop fishing.

**Table 10:** Quantitative results for fishery policy analysis.

	Number of trips	Fishing days per trip	Total travel distance (km)	Estimated trip costs ($)
Type 1: Regular trips only fished in WCPO	14,457	13.0	2,227	$25,409
Type 2: Regular trips fished between WCPO and EPO	2,436	14.6	3,416	$30,974
Type 3: Regular trips only fished in EPO	1,455	13.7	4,011	$31,537
Type 4: Affected trips in EPO during WCPO closures	320	14.0	3,894	$29,092
Type 5: Affected trips in WCPO during EPO closures	226	14.8	2,083	$30,298

## Conclusions and discussion

Fishing trip cost is an important element for evaluating economic performance of the fisheries, and assessing the impact of fisheries management alternatives. Using the sampled trip cost data for the two longline fisheries, this study presented a case study using parametric machine learning algorithms to build models for trip cost estimation. We showed that using GLM with ML (Lasso regularization and n-fold cross-validation techniques) was able to select model covariates with no multicollinearity and create models with good predictive power, so that individual trip costs could be estimated using trip and vessel-specific information of all fishing trips. This study showed that the new approach with GLM and ML provided a better fitting model when compared with the previous efforts in trip cost estimation for the Hawaii longline fishery that used OLS or OLS with log transformed trip costs, as bias correction was not required. In addition, the new modeling approach incorporated model prediction performance, which was not considered in previous trip cost modeling research, but it is an important performance metric to consider if we want to use the model to predict unsampled trip costs and conduct other economic applications.

Moreover, this study presented an empirical application of the estimated trip cost model to conduct a regulatory impact analysis, which is required by national standard guidelines of NOAA Fisheries. With the individual trip costs estimated by the Hawaii trip cost model, we were able to quantify the fishing cost differences for different subgroups of the fleet; those were affected by various policy regimes of area closures vs. those were not impacted. For example, an average 14% increase in trip costs was found due to the area closures in the WCPO. However, the impacts of EPO closures on trip costs were lower.

One potential use of the trip cost models is to predict individual trip costs using individual trip-specific fishing operation information and vessel-specific information, and compare with revenue to evaluate the net returns for individual trips and at subgroup and fleet-wide level. The estimated trip costs from the trip cost model provides important information for sustainable fisheries management when regulatory changes or other external factors affect subgroups of a fleet differently. In addition, the trip cost models estimated in this study can be used in different fishery studies such as ecosystem modeling by adding the dynamic analysis of trip costs in different vessels.

Another potential application of the Hawaii trip cost model is to examine the effects of trip costs from climate change. Evidence suggests that the Hawaii longline fishermen have changed their behavior over time and space in accordance with oceanographic variability. Particularly, the Hawaii deep-set longline fishery has migrated and expanded the fishing effort to the northeast of the main Hawaiian Islands in the third quarter of the year due to the vertical overlap of bigeye tuna’s preferred thermal habitat with the depth of the deep-set hooks. This migration of fishing effort potentially could affect the economic performance of the fleet as the fleet was traveling further to their fishing ground [37, 55]. If we could determine the climate change impact on travel distance and fishing days, the trip cost model could be used to estimate the impact of climate change on trip costs and the potential economic effects on the fleet could be evaluated.

It is important to note that there are some limitations on the model applications. External changes in the future may affect the structure of the trip cost models. For example, if there were a significant improvement in vessel technology that affect the fuel efficiency, this would change the relationship between vessel characteristics and trip costs. Thus, a new trip cost model may be needed to take into account the external changes. For model application to estimate trip costs in the future, since the cost functions were developed using the data adjusted to 2018 dollars, the predicted cost for the future years may need to be inflation adjusted to its current value. Nevertheless, this study provides a rather direct and robust modeling approach using fishing operational data and vessel characteristics that are commonly available to predict trip costs for the two most important commercial fisheries in the Pacific Island region.

## Supporting information

S1 Appendix Definition of predictors and methods to derive predictors. (PDF)

S2 Appendix Test for skewness and normality of trip cost distribution. (PDF)

S1 Table Exclusions of sample for analysis. (PDF)

## References

[1] Anderson JL, Anderson CM, Chu J, Meredith J, Asche F, Sylvia G, et al. The fishery performance indicators: a management tool for triple bottom line outcomes. PLoS ONE. 2015; 10: e0122809. doi: 10.1371/journal.pone.0122809 25946194 PMC4422616

[2] Lam V, Sumaila UR, Dyck A, Pauly D, Watson R. Construction and first applications of global cost of fishing database. ICES J Mar Sci. 2011; 68: 1996–2004. 10.1093/icesjms/fsr121

[3] Sala E, Mayorga J, Costello C, Kroodsma D, Palomares M, Pauly D, et al. The economics of fishing the high seas. Sci Adv. 2018; 4: eaat2504. doi: 10.1126/sciadv.aat2504 29881780 PMC5990315

[4] Prellezo R. Exploring the economic viability of a mesopelagic fishery in the Bay of Biscay. ICES J Mar Sci. 2019; 76: 771–779. 10.1093/icesjms/fsy001

[5] Seung CK, Waters EC. A review of regional economic models of fisheries management in the U.S. Mar Resour Econ. 2006; 21: 101–124. 10.1086/mre.21.1.42629497

[6] Sumaila UR, Cheung W, Dyck A, Gueye K, Huang L, Lam V, et al. Benefits of rebuilding global marine fisheries outweigh costs. PLoS ONE. 2012; 7: e40542. doi: 10.1371/journal.pone.0040542 22808187 PMC3396648

[7] Chae D, Pascoe S. Use of simple bioeconomic models to estimate optimal effort levels in the Korean coastal flounder fisheries. Aqua Living Res. 2005; 18: 93–101. 10.1051/alr:2005012

[8] Daigle RM, Monaco CJ, Elgin AK. An adaptable toolkit to assess commercial fishery costs and benefits related to marine protected area network design. F1000Research. 2017; 4: 1234. 10.12688/f1000research.7312.2 PMC5345778 28357034

[9] Lassen H, Pedersen SA, Frost H, Hoff A. Fishery management advice with ecosystem considerations. ICES J Mar Sci. 2013; 70: 471–479. 10.1093/icesjms/fss208

[10] Nielsen JR, Thunberg E, Holland DS, Schmidt JO, Fulton EA, Bastardie F, et al. Integrated ecological-economic fisheries models—Evaluation, review and challenges for implementation. Fish Fish. 2018; 19: 1–29. 10.1111/faf.12232

[11] Das C.Northeast trip cost data—overview, estimation, and predictions. 2013. US Dep. Commerce, NOAA Tech. Memo. NOAA-TM-NMFS-NE-227. 10.7289/V5571905

[12] Kalberg K, Pan M. 2012 Economic cost earnings of pelagic longline fishing in Hawaii. 2016. US Dep. Commerce, NOAA Tech. Memo. NOAA-TM-NMFS-PIFSC-56. 10.7289/V5/TM-PIFSC-56

[13] Davie S, Minto C, Officer R, Lordan C, Jackson E. Modelling fuel consumption of fishing vessels for predictive use. ICES J. Mar. Sci. 2015; 72: 708–719. 10.1093/icesjms/fsu084

[14] Chakravorty U, Nemoto K. Modeling the effects of area closure and tax policies: a spatial-temporal model of the Hawaii longline fishery. Mar Resour Econ. 2001; 15: 179–204. 10.1086/mre.15.3.42629301

[15] Daures F, Trenkel VM, Guyader O. Modelling the fishing costs of French commercial vessels in the Bay of Biscay. Fish Res. 2013; 146: 74–85. 10.1016/j.fishres.2013.03.022

[16] Li S, Pan M. Fishing opportunities under the sea turtle interaction caps—a spatial bio-economic model for the Hawaii-based longline swordfish. 2011. University of Hawaii, SOEST Publication 11–02, JIMAR Contribution 11–378. Available from: https://www.soest.hawaii.edu/PFRP/soest_jimar_rpts/li_pan_2011.final.pdf

[17] Dodd S, Bassi A, Bodger K, Williamson P. A comparison of multivariable regression models to analyse cost data. J Eval Clin Pract. 2006; 12: 76–86. doi: 10.1111/j.1365-2753.2006.00610.x 16422782

[18] Jia S, Rathi S. On predicting log-transformed linear models with heteroscedasticity. SAS Global Forum. 2008; 370: 1–6. Available from: https://support.sas.com/resources/papers/proceedings/pdfs/sgf2008/370-2008.pdf

[19] Manning WG, Mullahy J. Estimating log models: to transform or not to transform? J Health Econ. 2001; 20: 461–494. doi: 10.1016/s0167-6296(01)00086-8 11469231

[20] Frost C, Thompson SG. Correcting for regression dilution bias, comparison of methods for a single predictor variable. J R Statist Soc A. 2000; 196: 173–189. 10.1111/1467-985X.00164

[21] Wang L, Currans KM. Detransformation bias in non-linear trip generation models. J Urban Plan Dev. 2018; 144. doi: 10.1061/(ASCE)UP.1943-5444.0000477 30906108 PMC6425945

[22] Kirkpatrick JA, Benjamin S, DePiper G, Murphy T, Steinback S, Demarest C. Socio-economic impact of outer continental shelf wind energy development on fisheries in the U.S. Atlantic, Volume II-Appendices. US Dep. Interior, OCS Study BOEM 2017–012. 2017. Available from: https://espis.boem.gov/final%20reports/5581.pdf

[23] Agier L, Portengen L, Chadeau-Hyam M, Basagana X, Giorgis-Allemand L, Siroux V, et al. A systematic comparison of linear regression-based statistical methods to access exposome-health associations. Environ Health Perspect. 2016; 124: 1848–1856. doi: 10.1289/EHP172 27219331 PMC5132632

[24] James G, Witten D, Hastie T, Tibshirani R. An introduction to statistical learning with applications in R. New York: Springer; 2013.

[25] Mullainathan S, Spiess J. Machine learning: an applied econometric approach. J Econ Perspect. 2017; 31: 87–106. 10.1257/jep.31.2.87

[26] Witten IH, Frank E, Hall MA, Pal CJ. Data mining: practical machine learning tools and techniques, fourth ed. Cambridge: Morgan Kaufmann; 2017.

[27] Athey S, Imbens GW. Machine learning methods economists should know about. Annu Rev Econom. 2019; 11: 685–725. 10.1146/annurev-economics-080217-053433

[28] Tibshirani R. Regression shrinkage and selection via the Lasso. J R Stat Soc Series B Stat Methodol. 1996; 58: 267–88. 10.1111/j.2517-6161.1996.tb02080.x

[29] Januaviani T, Gusriani N, Joebaedi K, Supin S, Subiyanto. The best model of LASSO with the LARS (least angle regression and shrinkage) algorithm using Mallow’s C_p_. World Sci. News. 2019; 116: 245–252.

[30] Zhou X, Huang X. Reliability analysis of slopes using UD-based response surface methods combined with LASSO. Eng. Geol. 2018; 233: 111–123. 10.1016/j.enggeo.2017.12.008

[31] Desboulets LDD. A review of variable selection in regression analysis. Econometrics. 2018; 6: 1–27.

[32] Chen J, Hoogh K, Gulliver J, Hoffmann B, Hertel O, Ketzel M, et al. A comparison of linear regression, regularization, and machine learning algorithms to develop Europe-wide spatial models of fine particles and nitrogen dioxide. Environ Intl. 2019; 130: 194934. 10.1016/j.envint.2019.104934 31229871

[33] Morozova O, Levina O, Uuskula A, Heimer R. Comparison of subset selection methods in linear regression in the context of health-related quality of life and substance abuse in Russia. BMC Med Res Methodol. 2015; 15: 1–17. doi: 10.1186/1471-2288-15-1 26319135 PMC4553217

[34] Smith G. Step away from stepwise. J Big Data. 2018; 5: 1–12. 10.1186/s40537-018-0143-6

[35] Zhang H, Nettleton D, Zhu Z. Regression-enhanced random forests. JSM Proceedings 2017, Section on Statistical Learning and Data Science. Alexandria, VA: American Statistical Association. 636–647.

[36] Wood SN. Generalized additive model. An introduction with R. 2nd ed. Florida: Chapman and Hall; 2017.

[37] Woodworth-Jefcoats PA, Polovina JJ, Drazen JC. Synergy among oceanographic variability, fishery expansion, and longline catch composition in the central North Pacific Ocean. Fish Bull. 2018; 116: 228–239. 10.7755/FB.116.3.2

[38] Erauskin-Extramiana M, Arrizabalaga H, Hobday A, Cabre A, Ibaibarriaga L, Arregui I, et al. Large-scale distribution of tuna species in a warming ocean. Glob Chang Biol. 2019; 25: 2043–2060. doi: 10.1111/gcb.14630 30908786

[39] Dhurandhar A, Petrik A. Efficient and accurate methods for updating generalized linear models with multiple feature additions. J Mach Learn Res. 2014; 15: 2607–2627.

[40] H2O.ai. h2o: R Interface for H2O. R package version 3.30.0.60 [software]. 2020. Available from: https://github.com/h2oai/h2o-3

[41] Gholamy A, Kreinovich V, Kosheleva O. Why 70/30 or 80/20 relation between training and testing sets: a pedagogical explanation. 2018. Department Technical Report: UTEP-CS-18-09. Available from: https://scholarworks.utep.edu/cgi/viewcontent.cgi?article=2202&context=cs_techrep

[42] Boehmke B, Greenwell B. Hands-on machine learning with R. Florida: CRC Press; 2020. doi: 10.1038/s41467-020-19703-y

[43] Arlot S, Celisse A. A survey of cross-validation procedures for model selection. Statist Surv. 2010; 4: 40–79. 10.1214/09-SS054

[44] Kohavi R. A study of cross-validation and bootstrap for accuracy estimation and model selection. Proc. of the 14th Int. Joint Conference on Artificial Intelligence. 1995; 2: 1137–1143. Available from: http://citeseerx.ist.psu.edu/viewdoc/download;jsessionid=017123C57C62B8C3765D077D6B8D6C81?doi=10.1.1.133.9187&rep=rep1&type=pdf

[45] Kuhn M, Johnson K. Applied predictive modeling. New York: Springer; 2013.

[46] R Core Team. R: a language and environment for statistical computing. Version 1.2.5033 [software]. R Foundation for Statistical Computing. Vienna, Austria; 2019. Available from: https://www.R-project.org

[47] Pan M. Tracking changes on fisheries economic performance—continuous economic data collection programs for the Hawaii and American Samoa longline fisheries 2005–2016. 2018. US Dep. Commerce, NOAA Tech. Memo. NOAA-TM-NMFS-PIFSC-73. 10.25923/hqhf-d906

[48] Schau EM, Ellingsen H, Endal A, Aanondsen SA. Energy consumption in the Norwegian fisheries. J Clean Prod. 2009; 17: 325–334. 10.1016/j.jclepro.2008.08.015

[49] PIFSC, Fisheries Monitoring and Analysis Program. Hawaii longline logbook from 2005–2018. 2019a. National Marine Fisheries Service, Pacific Islands Fish Sci Cent. https://inport.nmfs.noaa.gov/inport/item/2721

[50] PIFSC, Fisheries Monitoring and Analysis Program. American Samoa longline logbook from 2006–2018. 2019b. National Marine Fisheries Service, Pacific Islands Fish Sci Cent. https://inport.nmfs.noaa.gov/inport/item/1775

[51] Ayers AL, Hospital J, Boggs C. Bigeye tuna catch limits lead to differential impacts for Hawaii longliners. Mar Policy. 2018; 94: 93–105. 10.1016/j.marpol.2018.04.032

[52] Richmond L, Kotowicz D, Hospital J. Monitoring socioeconomic impacts of Hawaii’s 2010 bigeye tuna closure: complexities of local management in a global fishery. Ocean Coast Manag. 2015; 106: 87–96. 10.1016/j.ocecoaman.2015.01.015

[53] Chan HL. Economic impacts of Papahānaumokuākea Marine National Monument expansion on the Hawaii longline fishery. Mar Policy. 2020; 115: 103869. 10.1016/j.marpol.2020.103869

[54] Mangi SC, Rodwell LD, Hattam C. Assessing the impacts of establishing MPAs on fishermen and fish merchants: the case of Lyme Bay, UK. AMBIO. 2011; 40: 457–468. doi: 10.1007/s13280-011-0154-4 21848135 PMC3357819

[55] Gilman E, Chaloupka M, Read A, Dalzell P, Holetschek J, Curtice C. Hawaii longline tuna fishery temporal trends in standardized catch rates and length distributions and effects on pelagic and seamount ecosystems. Aquat Conserv. 2012; 22: 446–488. 10.1002/aqc.2237

