# Supplementary material for: Fishing trip cost modeling using generalized linear model and machine learning methods – A case study with longline fisheries in the Pacific and an application in Regulatory Impact Analysis
Source: PLoS One. 2021 Sep 7;16(9):e0257027. doi: 10.1371/journal.pone.0257027 (PMC8423239; doi:10.1371/journal.pone.0257027)
Supplement: S1 Appendix — (PDF) [file pone.0257027.s001.pdf]

S1 Appendix. Definition of predictors and methods to derive predictors.

Number of fishing days is represented by the number of fishing sets per trip because a set is usually completed within a day. Trip length is the number of days between departure date and return date. Fishing set and haul locations are available in the logbooks. Travel distance to fishing ground is defined as the travel distance from the departure port to the first fishing set and haul locations (average of begin set, end set, begin haul, and end haul locations), and the travel distance from the last fishing set and haul locations to the returning port. These two distances were added and divided by two to get the average travel distance to fishing ground. Total travel distance was calculated the same as the travel distance to fishing ground, plus the travel distance between all individual fishing set and haul locations. Travel distance was calculated using geosphere package (Hijmans et al. 2017) in Program R. Monthly fuel price for Hawaii was from American Automobile Association (DBEDT 2019). We used monthly fuel price (based on the departure month of the trip) for the Hawaii models because the average trip length is about a month: 22 days for deep-set trips and 32 days for shallow-set trips. Annual fuel price for American Samoan models was calculated from the American Samoa longline trip cost data because their monthly fuel price was not available.

DBEDT. Monthly energy trends. 2019. https://dbedt.hawaii.gov/economic/energy-trends-2/.

Hijmans R J, Williams E, Vennes C. Geosphere: spherical trigonometry. R package version 1.5-7 [software]. 2017. Available: https://CRAN.R-project.org/package=geosphere.
