# Supplementary material for: Fishing trip cost modeling using generalized linear model and machine learning methods – A case study with longline fisheries in the Pacific and an application in Regulatory Impact Analysis
Source: PLoS One. 2021 Sep 7;16(9):e0257027. doi: 10.1371/journal.pone.0257027 (PMC8423239; doi:10.1371/journal.pone.0257027)
Supplement: S2 Appendix — (PDF) [file pone.0257027.s002.pdf]

S2 Appendix. Test for skewness and normality of trip cost distribution.

Table S3 shows the skewness and tests for skewness and normality for total trip cost distribution in Hawaii and American Samoa. The skewness value is greater than 1 for Hawaii trip costs and close to 1 for American Samoa trip costs, indicating trip costs are skewed right. The *t*-statistic of skewness for trip costs in both fisheries are greater than 1.96, rejecting the null hypothesis of no skew (Cramer and Howitt 2004). The Shapiro-Wilk test for normality shows that the *p* values are less than 0.05 for both trip costs, rejecting the null hypothesis of normality.

S3 Table. Skewness and Test of Skewness and Normality of Total Trip Costs in Hawaii and American Samoa Longline Fisheries.

	Hawaii	American Samoa
Skewness	1.279	0.990
Standard error of skewness	0.047	0.195
t-statistics of skewness	27.213	5.077
Shapiro-Wilk test	0.913	0.933
Shapiro-Wilk test significance	0.000	0.000
N	2,746	155
