# Supplementary material for: Fishing trip cost modeling using generalized linear model and machine learning methods – A case study with longline fisheries in the Pacific and an application in Regulatory Impact Analysis
Source: PLoS One. 2021 Sep 7;16(9):e0257027. doi: 10.1371/journal.pone.0257027 (PMC8423239; doi:10.1371/journal.pone.0257027)
Supplement: S1 Table — (PDF) [file pone.0257027.s003.pdf]

S1 Table. Exclusions of sample for analysis.

	Hawaii cost data	American Samoa cost data
Total observed sample	2,948	164
Missing values		
Fuel use or fuel cost	95	-
Gear cost	10	-
Communication cost	10	-
Provision cost	3	-
Lightsticks cost	1	-
Outliers*		
Fuel used too high	4	3
Fuel used too low	15	3
Fuel price too high	14	-
Fuel price too low	11	-
Lightsticks cost too high	6	-
Lightsticks cost too low	2	-
Oil cost too high	4	1
Bait cost too high	3	1
Communication cost too high	3	-
Gear cost too high	2	-
Freon & other freezer cost too high	-	1
No matching logbook data	19	-
Final sample for modeling	2,746	155

Note. *Outliers were checked by examining the total value per trip and average value per trip-day for each individual cost items. Depending on the distribution of individual cost items, Hawaii cost items with values above the 95th or 99th percentile or values below the 5th or 1st percentile were considered outliers. For American Samoa cost data, outliers included cost items with values above the 99th percentile or below the first percentile.
